# The role of NLRP3 inflammasome-mediated pyroptosis in ischemic stroke and the intervention of traditional Chinese medicine

**DOI:** 10.3389/fphar.2023.1151196

**Published:** 2023-04-21

**Authors:** Jia-Xin Long, Meng-Zhi Tian, Xiao-Yi Chen, Huang-He Yu, Huang Ding, Fang Liu, Ke Du

**Affiliations:** ^1^ School of Pharmacy, Hunan University of Chinese Medicine, Changsha, China; ^2^ College of Integrated Traditional Chinese Medicine and Western Medicine, Hunan University of Chinese Medicine, Changsha, China

**Keywords:** ischemic stroke (IS), pyroptosis, NLRP3, pathways, traditional Chinese medicine (TCM)

## Abstract

Ischemic stroke (IS) is the second leading cause of death and disability in the world. Pyroptosis, a form of programmed cell death initiated by caspases, participates in the occurrence and development of IS. Because it can increase cell membrane permeability, mediate the release of inflammatory factors, and aggravate inflammation, inhibiting this process can significantly reduce the pathological injury of IS. The nucleotide binding oligomerization domain-like receptor family pyrin domain protein 3 (NLRP3) is a multiprotein complex whose activation is the core link of pyroptosis. In recent years, studies have reported that traditional Chinese medicine (TCM) could regulate pyroptosis mediated by NLRP3 inflammasome through multi-channel and multi-target networks and thus exert the effect against IS. This article reviews 107 papers published in recent years in PubMed, Chinese National Knowledge Infrastructure (CNKI), and WanFang Data in recent years. It has found that the activation factors of NLRP3 inflammasome include ROS, mitochondrial dysfunction, K^+^, Ca^2+^, lysosome rupture, and trans-Golgi breakdown. TLR4/NF-κB/NLRP3, ROS/TXNIP/NLRP3, AMPK/Nrf2/NLRP3, DRP1/NLRP3, TAK1/JNK/NLRP3 signaling pathways regulate the initiation and assembly of the NLRP3 inflammasome, subsequently induce pyroptosis, affecting the occurrence and development of IS. TCM can affect the above signaling pathways and regulate the pyroptosis mediated by NLRP3 inflammasome, so as to play a protective role against IS, which provides a new entry point for discussing the pathological mechanism of IS and a theoretical basis for developing TCM treasure house.

## Introduction

An analysis of global disease systems in *Lancet Neurology* shows that ischemic stroke (IS) is the second leading cause of mortality and disability worldwide, and the economic costs of its treatment and post-stroke care are substantial (GBD, 2016 Stroke Collaborators, 2019). IS is a clinical syndrome of neurological damage caused by cerebral blood supply disorder, brain tissue hypoxia, sugar deficiency, and tissue necrosis (Zhao et al., 2022). In recent years, a new type of programmed cell death called pyroptosis, which is closely related to inflammation, has been discovered (Tang et al., 2019). The 2002 Nobel Prize in Physiology or Medicine and the Nobel Prize in Chemistry in 2004 were awarded to scientists who have made pioneering contributions in the field of pyroptosis. The research indicates that pyroptosis has the characteristics of both apoptosis and necrosis, showing that the nuclear morphology is complete and the cell membrane is broken, leading to the release of cell contents and causing inflammation (Wang et al., 2017). Nucleotide-binding oligomerization domain-like receptor protein 3 (NLRP3) inflammasome is the key protein of pyroptosis and closely participates in the process of pyroptosis (Huang et al., 2021). Recent studies have found that pyroptosis mediated by NLRP3 inflammasome participates in the pathological process of IS (Fu et al., 2020; McKenzie et al., 2020). Kind of literature suggests that acupuncture, traditional Chinese medicine monomer, traditional Chinese medicine compound, and Chinese patent medicine can regulate NLRP3 inflammasome-mediated pyroptosis related signaling pathways to play a neuroprotective role in IS (Wang M et al., 2020; Ran et al., 2021; Chen et al., 2022). This article will elucidate the activation mechanism of NLRP3 inflammasome in the central nervous system (CNS), and review the research on the intervention of traditional Chinese medicine on NLRP3 inflammasome.

## NLRP3 inflammasome in central nervous system and activation mechanism

The NLRP3 inflammasome is a multi-protein complex composed of NLRP3, ASC and pro-cassase-1, which plays an important role in the classical pyroptosis pathway (Lu et al., 2020). In 2004, *Immunity* first reported that NLRP3 inflammasomes are the basic molecules related to auto-inflammation (Agostini et al., 2004). In 2018, *Nature* further reported the activation mechanism of NLRP3 inflammasome, that is, the disbanded trans-Golgi network activated inflammasome by inducing the transport and aggregation of NLRP3 through phospholipid PtdIns4P (Chen and Chen, 2018). The activation of the NLRP3 inflammasome produces caspase-1. Subsequently, caspase-1 cleaves and splits gasdermin D (GSDMD) and pro-interleukin-1β (pro-IL-1β), pro-interleukin-18 (pro-IL-18), forming GSDMD-N and IL-1β, IL-18 (Chen et al., 2018; Alishahi et al., 2019). Then GSDMD-N acts on phospholipid molecules on the cell membrane to form pores, leading to cell osmotic swelling, cell membrane rupture and pyroptosis, simultaneously IL-1β and IL-18 are released out, which expands the local inflammatory response (Chen S et al., 2018). NLRP3 widely exists in neurons, microglia and cerebral vascular endothelial cells. The activation of NLRP3 inflammasome proceeds in two steps. The first step is priming. The second step is the assembly of inflammasome, which is a step of many studies on activation. Multiple upstream signals can induce the formation of the inflammasome by activating the oligomerization of NLRP3 protein.

### Priming of NLRP3 inflammasome

**FIGURE 1:**
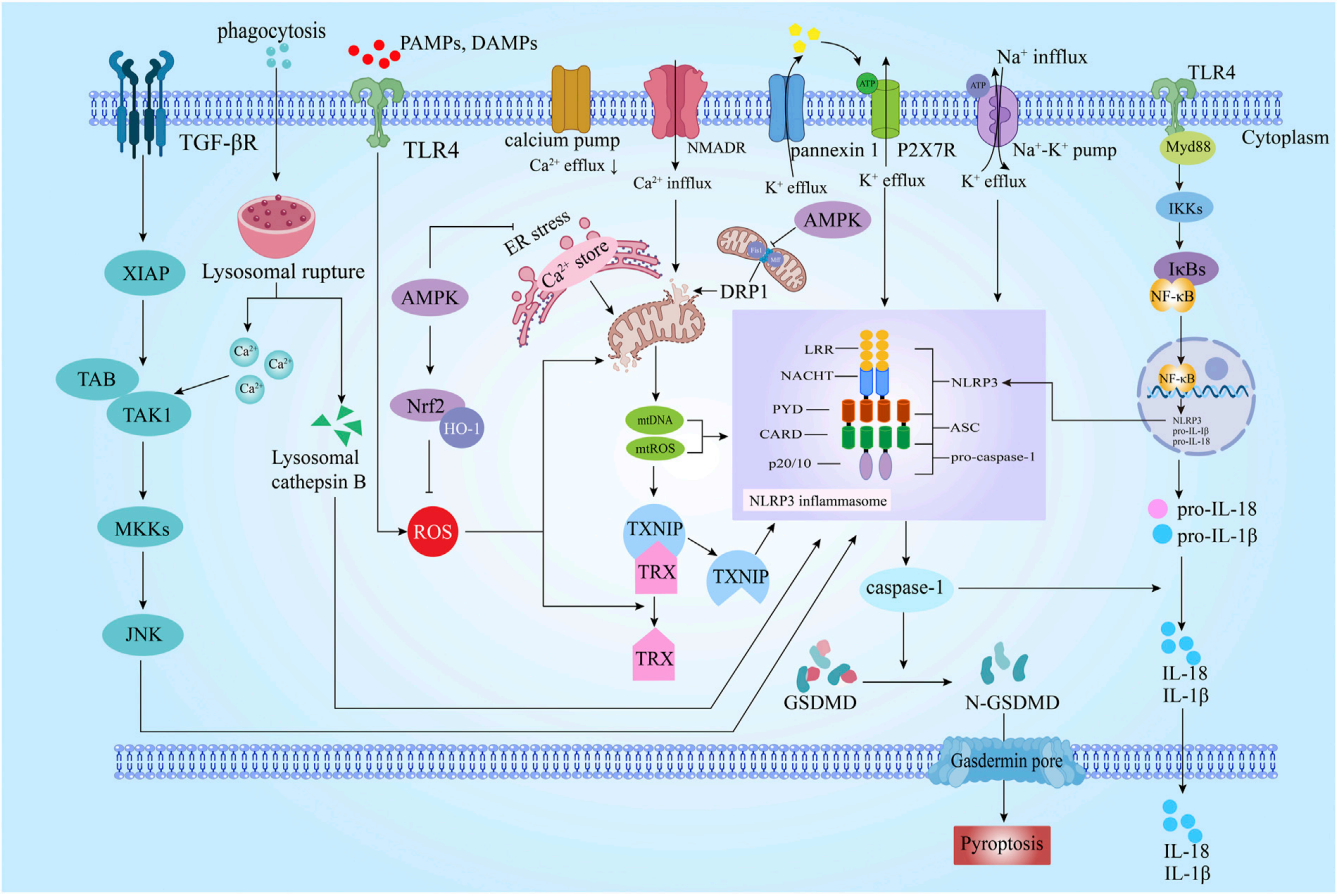
Schematic diagram of the molecular mechanism of pyroptosis in ischemic stroke (IS). Pyroptosis is a kind of programmed cell death process mediated by nucleotide-binding oligmerization domain-like receptor protein 3 (NLRP3) inflammasome and activated by Caspase-1) . It has the characteristics of both apoptosis and necrosis NLRP3. The initiation of the NLRP3 inflammasome involves a series of signaling pathways, such as TLR4/NF-κB/NLRP3, ROS/TXNIP/NLRP3, AMPK/NLRP3, DRP1/NLRP3, TAK1/JNK/NLRP3. TGFβ: transforming growth factor-β; XIAP: X-linked inhibitor of apoptosis protein; TAK1: transforming growth factor-beta activated kinase 1; TAB: transforming growth factor-β activated kinase 1 binding protein; MKKs: mitogen-activated protein kinase kinase; JNK: c-JunN-terminalkinase; DAMPs: damage associated molecular pattern; PAMPs: pathogen associated molecular patterns; ROS: Reactive oxygen species; AMPK: AMP-activated protein kinase; Nrf2: nuclear factor erythroid 2-related factor 2; HO-1: Heme Oxygenase-1; ER: endoplasmic reticulum; TXNIP: TRX-interacting protein; TRX: thioredoxin; mtROS: mitochondrial ROS; mtDNA: mitochondrial DNA; DRP1: dynamin-related protein 1; NMDAR: Nmethyl-D-aspartate receptor; ASC: apoptosis-associated speck-like protein containing acaspase recruitment domain; LRR: leucine-rich repeats; NACHT: nucleotidebinding and oligomerization; PYD: pyrin domain; CARD: Caspaseact ivat ing recruitment do main; caspase-1: cysteinyl aspartate specific proteinase-1; TLR4: toll-like receptor 4; MyD88: myeloid differentiation factor 88; IKKs: IkappaB kinases; IκBs: I kappaBs; NF-κB: nuclear factor kappa B; GSDMD: Gasdermin D.

Damage-associated molecular patterns (DAMPs) and pathogen-associated molecular pattern ligands (PAMPs) generated by cerebral ischemia and hypoxia cross the blood-brain barrier (BBB) into the CNS and activate the Toll-like receptor 4 (TLR4) on the surface of neurons, microglia, and astrocyte cell membranes (Shi et al., 2019). TLR4 is a transmembrane receptor protein involved in innate immune response (Li L et al., 2021), which can detect the danger signals of the extracellular environment. TLR4 initiates a signaling cascade to produce inflammatory cytokines (TNF, IL-12, IL-12, IL-6, IL-8, IL-1β, etc.). The key signal ligand downstream of TLR4 is myeloid differentiation factor 88 (MyD88). TLR4 and MyD88 promote the activation of tumor necrosis factor receptor-associated factor 6 (TRAF6) and activate the downstream transcriptional regulator nuclear factor κB (NF-κB) (Mitchell et al., 2018). NF-κB activation increases the transcription of NLRP3 inflammasome related protein coding gene (Jin et al., 2019), thereby activating caspase-1 and isolating the N and C ends of GSDMD to induce pyroptosis (Mohamadi et al., 2018) (Figure 1).

Ye et al. (2019) established an adult male C57BL/6J wild-type mouse middle cerebral occlusion/reperfusion (MCAO/R) *in vivo*. Immunofluorescence staining and western blot showed that the expression of NLRP3 inflammasome and their related proteins in neurons and microglia was activated. Subsequently, an *in vitro* oxygen-glucose deprivation/reperfusion (OGD/R) model was established in HT22 and BV2 cells. Results showed TLR4/NF-κB was significantly upregulated, NLRP3 inflammasomes were activated and M1 microglia/macrophages were polarized (Ye et al., 2019). Liu et al. (2021) used the TLR4 inhibitor TAK242 to reverse prove that the TLR4/NF-KB/NLRP3 pathway was activated in OGD/R BV2 cells. Wang K et al. (2020) detected pyroptosis in the ischemic cortex by dUTP nick end labeling (TUNEL) measurement and lactate dehydrogenase (LDH) release, and detected NLRP3 inflammasome assembly and inflammatory cytokine secretion by enzyme-linked immunosorbent assay (ELISA) and western blot. Confirming the activation of the TLR4/NF-κB/NLRP3 pathway induced by MCAO. While inhibiting NF-κB/NLRP3 protects neurons from OGD-induced pyroptosis (Kang et al., 2021). The above research shows that pyroptosis mediated by TLR4/NF-κB/NLRP3 pathway has a negative regulatory effect on IS, and targeted inhibition of this pathway plays a protective role.

### Assembly of NLRP3 inflammasome

#### NLRP3 inflammasome assembly induced by reactive oxygen species (ROS)

All known PAMPs and DAMPs trigger the production of ROS, which can then induce the assembly of the NLRP3 inflammasome. High levels of ROS promote the dissociation of the oxidation-reduction sensitive signal complex TXNIP, which is translocated from the nucleus to the cytoplasm (Luo et al., 2022). TXNIP binds to the NLRP3 receptor domain (mainly LRR domain), thus inducing the activation of NLRP3 inflammasome (Chen D et al., 2018). *In vivo* experiments show that ROS drives TXNIP overexpression in MCAO rats and MCAO/R C57BL/6 mice, then TXNIP aggravates brain damage through redox imbalance, subsequently activates NLRP3 inflammasome, caspase-1, and causes the release of IL-1β (Cao et al., 2016; Zeng et al., 2021; Yao et al., 2022). *In vitro* experiments show that ROS/TXNIP/NLRP3 signaling pathway activation induces pyroptosis in OGD/R and OGD primary cortical neurons (Yao et al., 2022). Knockdown of TXNIP significantly decreased the expression of NLRP3 in OGD-induced neurons (Liu et al., 2020). The above results suggest that ROS/TXNIP mediated activation of NLRP3 inflammasome is a key factor in IS.

The AMP-activated protein kinase (AMPK)/NF-E2 related factor 2 (Nrf2) pathway suppresses the assembly of NLRP3 inflammasome through anti-ROS. AMPK is phosphorylated and activated in response to an increase in the intracellular AMP/ADP ratio during ischemia and glycogen deprivation (Gu et al., 2018). AMPK directly phosphorylates the Nrf2 with an endogenous neuroprotective effect (Joo et al., 2016; Yu H et al., 2020). The activated Nrf2 translocates from the cytoplasm to the nucleus and binds to the antioxidant response element (ARE) (Fuse and Kobayashi, 2017). Hou et al. (2018) intraperitoneally injected tert-butylhydroquinone (tBHQ) to activate Nrf2. Western blot and qRT-PCR results showed that NLRP3 inflammasome and downstream caspase-1, IL-18, and IL-1β were significantly reduced, whereas Nrf2 knockout produced the opposite result. Transfection of Nrf2 into mice with inflammation had the same effect (Liu et al., 2017). When MCAO rats were treated with AMPK inhibitor dorsomorphin, immunofluorescence and western blot results showed that microglia/macrophages were activated, p-AMPK and Nrf2 were decreased, and NLRP3 was upregulated (Yu J et al., 2020). This is consistent with the result of knocking out Nrf2 (Liu et al., 2017), indicating that Nrf2 could inhibit the expression of NLRP3 (Liu et al., 2017). The downregulation of Nrf2 can also promote the pyroptosis of vascular endothelial cells induced by NLRP3 (Hu et al., 2018). The above experimental results suggest that up-regulating the expression of AMPK or Nrf2 can significantly improve pyroptosis, promote neural function recovery and improve IS (Qiu et al., 2016).

#### NLRP3 inflammasome assembly induced by mitochondrial dysfunction

Mitochondrial dysfunction is an important feature of IS pathophysiology (Hasan et al., 2021). DRP1 is a protein related to mitochondrion division which was originally mainly distributed in the cytoplasm of cells. It was recruited by some signals and translocated to the outer membrane of mitochondria to form helical oligomers, which caused mitochondrion division and aggravated mitochondrial dysfunction (Duan et al., 2020). Phosphorylation of DRP1 reduces mitochondrial fission by regulating its translocation via AMPK (Hu et al., 2017). After activation, DRP1 combines with Fission1 (Fis1) and mitochondrial fission factor (Mff) to mediate the metabolic disorder of cells and inhibit glutathione in mitochondria to weaken the ability of free radical scavenging, further increase mitochondrial reactive oxygen species (mtROS), thus up-regulating the level of NLRP3 protein and producing IL-1β, cause pyroptosis, and eventually cause ischemic damage to neurons (Park et al., 2018; Kleele et al., 2021). Knockout of DRP1 can improve the function of mitochondria and reduce the level of NLRP3 protein to reduce pyroptosis (Zhong et al., 2022). It was found that in MCAO/R rats and OGD SH-SY5Y cells, DRP1 translocated mitochondria, resulting in the mitochondrial division, mitochondrial dysfunction, and then produced a large number of ROS, activated NLRP3 inflammasome, and induced pyroptosis (Guo et al., 2018; Hu et al., 2020). Inhibition of the DRP1 phosphorylation cannot only protect the integrity of mitochondria, but also reduce the activation of NLRP3 inflammasome and reduce pyroptosis (Zhou et al., 2011; Carneiro et al., 2012). Therefore, downregulation of the DRP1/NLRP3 pathway can effectively improve mitochondrial damage, inhibit pyroptosis and play a neuroprotective role.

#### NLRP3 inflammasome assembly induced by ionic steady-state imbalance

Intracellular K^+^ efflux is a key factor in the activation of NLRP3 inflammasome (Muñoz-Planillo et al., 2013). K^+^ efflux leads to the interaction between the inactive NLRP3 positive motif and the negative PIP on the Golgi membrane, which causes the local accumulation of NLRP3 and provides sufficient conditions for the activation of NLRP3 inflammasomes. The researchers found that NLRP3 inflammasomes could be activated without decreasing the intracellular K^+^ concentration in the early stage of crystal stimulation, indicating that the activation of NLRP3 activation signal was not dependent on K^+^ efflux, which denied the necessity of K^+^ efflux for the activation of NLRP3 inflammasomes (Zhang et al., 2018). Therefore, the reduction of intracellular K^+^ concentration provides sufficient and unnecessary conditions for the activation of NLRP3 inflammasomes. Both ATP synthesis and Na^+^- K^+^- ATPase activity are reduced during IS, leading to an increase in Na^+^ influx and K^+^ efflux, thus reducing the intracellular K^+^ (Zhu et al., 2022). ATP released by necrotic cells binds to the P2X4 receptor, causing the receptor to open, leading to K^+^ efflux. In addition, the necrotic cells passively release K^+^, resulting in high extracellular K^+^. K^+^ activates the pannexin 1 channel on the cell membrane, further releasing ATP. Previous studies have revealed that P2X7R triggers the second stage of assembly and activation of NLRP3 inflammasomes (Albalawi et al., 2017), which can activate NLRP3 inflammasomes in astrocytes and participate in the pathogenesis of IS (Ye et al., 2017).

Ca^2+^ influx is another important factor independent of the activation of NLRP3 inflammasomes induced by K^+^ efflux (Katsnelson et al., 2015). Ca^2+^ influx is another important factor in NLRP3 inflammasome activation. There are four main ways to cause cytoplasm Ca^2+^ overload in IS. The first is that the N-methyl-D-aspartate receptor (NMDAR) of the postsynaptic membrane is overexcited, which mediates the opening of its coupled calcium channel, and a large amount of extracellular Ca^2+^ influx (Ludhiadch et al., 2022). The second is the release of Ca^2+^ from the endoplasmic reticulum cavity to the cytoplasm caused by endoplasmic reticulum stress (ERS) (Groenendyk et al., 2021). The third is that Ca^2+^ in lysosomes is released to the cytoplasm through the non-selective cation channel TRPML (Weber and Schilling, 2014). The last one is that the permeability of plasma membrane-related calcium pump is reduced due to the lack of ATP activity, leading to the reduction of Ca^2+^ transfer to the outside cell (Hu and Song, 2017). At present, there are two opinions about the Ga^2+^ in the activation of NLRP3 inflammasome. One is that Ca^2+^ is directly involved in the activation of the NLRP3 inflammasome, because the increase of Ca^2+^ can promote the interaction between NLRP3 and ASC (Lee et al., 2012). Another opinion is that the increase of the excessive release of Ca^2+^ from the endoplasmic reticulum (ER) leads to an overload of mitochondrial Ca^2+^, leading to mitochondrial dysfunction and activation of NLRP3 inflammasome (Murakami et al., 2012). No matter whether Ca^2+^ directly or indirectly activates the NLRP3 inflammasome, it is clear that Ca^2+^ overload induces pyroptosis in IS (Wang L et al., 2020). Interestingly, *Science* published a research article related to the mechanism of Ca^2+^ activating pyroptosis in 2018. It was found that Ca^2+^ influx through the GSDMD pore was used as a signal for cells to start membrane repair, and ESCRT complexes required for transport were recruited to repair damaged membrane systems. Since the inhibition of ESCRT-III could significantly improve pyroptosis, this article provides new insight into the endogenous mechanism of pyroptosis (Rühl et al., 2018).

#### NLRP3 inflammasome assembly induced by lysosome rupture

The lysosomal membrane loses its stability, integrity and permeability, and lysosome membrane permeabilization (LMP) occurs after IS, leading to the release of cathepsin B and Ca^2+^ (Bruchard et al., 2013; Repnik et al., 2014). Various cathepsins play a role in pro-synthesis and NLRP3 activation (Orlowski et al., 2015). It is reported that the release of lysosomal cathepsin B is IL-1β release required in *Nature* (Duewell et al., 2010). Pharmacological inhibition of cathepsin activity or genomic deletion of various cathepsin can significantly reduce the signal transduction of NLRP3 inflammasome. For example, cathepsin B inhibitor CA-074-Me could partially inhibit the activation of NLRP3 inflammasome (Bruchard et al., 2013). However, there are also studies showing that gene deletion or knockdown of cathepsin B has little effect on NLRP3 inflammasome activation (Orlowski et al., 2015). Therefore, whether lysosomal cathepsin B is a sufficient and necessary condition for the activation of NLRP3 inflammasome is controversial. Ca^2+^ released by lysosome rupture is activated by NLRP3 inflammasomes via transforming growth factor-beta activated kinase 1 (TAK1)/c-Jun NH2 terminal kinase (JNK) pathway. Ga^2+^-CaMKII promotes the binding of TAK to TAK 1 binding protein (transforming growth factor-β activated kinase 1 binding protein, TAB), so that phosphorylation of TAK1 activates downstream MAPK kinase (MAPK kinase, MAPKK) MKK4/7, and subsequently MKK4/7 specifically activates JNK (Hirata et al., 2017). JNK promotes the activation of the NLRP3 inflammasome by regulating ASC oligomerization (Hara et al., 2013). Zhang and colleagues confirmed that TAK1/JNK pathway is involved in the pathogenesis of IS in MCAO/R rats and OGD/R primary cortical neurons (Zhang Z et al., 2022). CNS extracellular pathological signal molecule lysophosphatidic acid (LPA) induced PC12 pyroptosis is related to JNK (Choi and Chun, 2013; Li and Zhang, 2020). In addition, lysosome rupture will also trigger a significant reduction of cytoplasmic K^+^ before NLRP3 inflammasome assembly and caspase-1 production, and is not related to NLRP3 inflammasome assembly and accumulation (Muñoz-Planillo et al., 2013). This indicates that lysosome rupture activates plasma membrane cation channel, which is a key early signal of NLRP3 inflammasome assembly (Katsnelson et al., 2015). Some studies have also shown that under certain experimental conditions, the effect of lysosome rupture on activating NLRP3 inflammasome signal may be very low. Therefore, lysosome rupture determines whether to activate or inhibit NLRP3 inflammasomes according to different cell environments.

## The intervention of traditional Chinese medicine on NLRP3 inflammasome-mediated pyroptosis in ischemic stroke

Modern pharmacological studies have found that most of the traditional Chinese medicine aims to prevent and treat IS by inhibiting the activation of NLRP3 inflammasomes and caspase-1, and by regulating its upstream related signal pathways, such as TLR4/NF-κB, ROS/TXNIP, AMPK/Nrf2 or DRP1/NLRP3 to inhibit the occurrence of pyroptosis. At the same time, it is also found that the same monomer or compound prescription can regulate and control the above multiple pathways to improve NLRP3-mediated pyroptosis. Thus, the influence of traditional Chinese medicine on pyroptosis anti-IS is characterized by multiple pathways.

### Acupuncture

Acupuncture is recommended by the World Health Organization (WHO) as an alternative strategy for IS treatment and nursing. Acupuncture can effectively improve IS by promoting nerve regeneration, improving blood flow in the infarct area, fighting apoptosis, and regulating neurochemicals (Chavez et al., 2017; Xu et al., 2018). In recent years, studies have found that acupuncture plays a neuroprotective role by inhibiting NLRP3 inflammasome-mediated pyroptosis (Zhang T et al., 2021; Chen et al., 2022). For example, acupuncture can protect MCAO rats by up-regulating the expression of SIRT1, inhibiting the activation of NLRP3 inflammasome and down-regulating the expression of IL-18 (Zhou et al., 2022). Xu et al. (2020) treated the MCAO/R rats with electroacupuncture at the four points of Zusanli, Sanyinjiao, Chize, and Hegu, and found that the infarction volume of the electroacupuncture group was reduced compared with the model group, and caspase-1 mRNA was lower. Liu et al. (2019) intervened in MCAO/R rats with eye acupuncture. Intervention of MCAO/R rats with eye acupuncture has no significant difference from that of the NLRP3 inflammasome inhibitor glibenclamide group. Both of them can inhibit the expression of P2X7R, NLRP3, pro-capase-1, ASC, caspase-1 protein in rats, indicating that eye acupuncture can inhibit the occurrence of pyroptosis. The results show that eye-acupuncture could inhibit the expression of P2X7R, NLRP3, pro-caspase-1, ASC, and caspase-1 proteins in rats, which is consistent with the trend of the group using NLRP3 inflammasome inhibitor glibenclamide, indicating that eye acupuncture could inhibit the occurrence of pyroptosis. miRNA-mediated pyroptosis is also involved in the development of IS. For example, the level of miR-223 in the cortex around the infarction of MCAO rats treated with electroacupuncture increased, while the level of NLRP3, caspase-1, IL-1β and IL-18 decreased (Sha et al., 2019). To sum up, acupuncture can regulate IS with multiple targets, which may become a new treatment tragedy for IS. However, a minority of the above studies have not carried out specific signal pathway studies, so the protective mechanism of acupuncture on neuronal damage after IS still needs to be confirmed by further research (Table 1).

**TABLE 1:** Advance in treatment of traditional Chinese medicine against IS by drug intervention pyroptosis.

Treatment	Traditional Chinese medicine	Model	Pathways	References
acupuncture	acupuncture	MCAO/R rats	SIRT1/NLRP3	Zhou et al. (2022)
	electroacupuncture	MCAO/R rats	not clear	Xu et al. (2020)
	eye acupuncture	MCAO/R rats	not clear	Liu et al. (2019)
effective components	Artesunate (ART)	MCAO/R rats	TLR4/NF-κB/NLRP3	Chen et al. (2021)
				
	Mulberroside A	OGD/R rat cortical neurons	TLR4/NF-κB/NLRP3	Wang et al. (2014)
	Carthamin yellow (CY)	MCAO/R rats	TLR4/NF-κB/NLRP3	Guo et al. (2021)
	Curcumin	MCAO/R rats, LPS with ATP induced-microglia pyroptosis, glutamate induced-SH-SY5Y cells neurotoxicity	TLR4/NF-κB/NLRP3, ROS/TXNIP/NLRP3	Li et al. (2015), Ran et al. (2021)
	Umbelliferone (UMB)	MCAO/R rats	ROS/TXNIP/NLRP3	Wang et al. (2015)
	Ginsenoside Rd (Rd)	MCAO/R rats, OGD/R cortical neuron	ROS/TXNIP/NLRP3	Yao et al. (2022)
	Irisin	MCAO mice, OGD PC12 cells	ROS/NLRP3, AMPK/NLRP3	Li et al. (2017), Peng et al. (2017)
	Ruscogenin	OGD/R-injured mouse brain microvascular endothelial cells (bEnd.3)	ROS/NLRP3, AMPK/NLRP3	Cao et al. (2016)
	Z-Guggulsterone (Z-GS)	MCAO rats; OGD cortical neuron	ROS/NLRP3, AMPK/NLRP3	Liu et al. (2020)
	Sinomenine (SINO)	MCAO mice OGDastrocyte/microglia cells	AMPK/NLRP3	Qiu et al. (2016)
	Astragalode IV (AS-IV)	MCAO rats, OGD/R SH-SY5Y cells	AMPK/NLRP3	Xiao et al. (2021)
	Resveratrol (Res)	LPS- and ATP-induced N9 microglial cells	AMPK/NLRP3	Tufekci et al. (2021)
	Hispidulin	MCAO/Rrats, OGD/R primary cerebral astrocytes cells	AMPK/NLRP3	An et al. (2019)
	Chrysophanol	tMCAO mice	NLRP3	Zhang et al. (2014)
	Quercetin	ECs	ROS/TXNIP/NLRP3	Wu et al. (2014)
	luteolin	ECs	ROS/TXNIP/NLRP3	Wu et al. (2014)
	epigallocatechin gallate	ECs	ROS/TXNIP/NLRP3	Wu et al. (2014)
	Dihydromyricetin (DHM)	ECs	Nrf2/NLRP3	Hu et al. (2018)
compound prescription	Angelica sinensis and Cinnamomum cassia (AC)	pMCAO rats, LPS-induced BV2 cells pyroptosis	TLR4/NF-κB/NLRP3	Luo et al. (2021)
	Renshen Shouwu (RSSW)	MCAO rats	TLR4/NF-κB/NLRP3	Li et al. (2020)
	Taohong Siwu decoction (THSWD)	MCAO/R rats	DRP1/NLRP3, TLR4/NF-KB/NLRP3, JNK/p38MAPK	Wang M et al., 2020; Zhou (2021)
	Panax ginseng and Angelica sinensis (CPA)	tMCAO rats; OGD/R BV2microglia cells	DRP1/NLRP3	Hu et al. (2020)
	Qingkailing injection (QKL)	MCAO/R rats	AMPK/NLRP3	Ma et al. (2019)
	Naoxinqing Capsule	MCAO/R rats	not clear	Dongyu et al. (2020)
	Shennaofuyuan Decoction (SNFYD)	OGD PC12 cells	not clear	Li (2019)

### Traditional Chinese medicine monomer

Traditional Chinese medicine monomer is an active ingredient extracted from traditional Chinese medicine. These traditional Chinese medicine monomers are mainly extracted by alcohol, supplemented by ultrasonic cracking (Wang et al., 2014; Yue. et al., 2016). Some TCM monomers have been approved by the China Medical Products Administration (NMPA) for the treatment of IS. In recent years, it has been found that many traditional Chinese medicine monomer can play an anti-pyroptosis role by regulating the signal pathway upstream of NLRP3 inflammasome. Mulberroside A is considered to be the main active ingredient of Cortex Mori, which shows extensive benefits in the routine oral water administration route (Wang et al., 2011). Mulberroside A can inhibit TLR4/NF-κB induced by OGD/R cortical neurons (Wang CP et al., 2014). Artesunate (ART), a derivative of artemisinin (Chen et al., 2021), can reduce the score of neurological deficits induced by MCAO/R rats, improve brain edema, and reduce the volume of cerebral infarction. Its mechanism is related to down-regulating TLR4/NF-κB/NLRP3 pathway (Ran et al., 2021). Carthamin yellow (CY) is a flavonoid compound isolated from safflower*,* it is considered that CY improves blood circulation and alleviates pain. Thus, CY is used for the treatment of cerebrovascular disease (Lu QY et al., 2019). Its protection mechanism is the same as that of ART (Guo et al., 2021). Curcumin is a natural polyphenolic compound in *Curcuma longa.* Curcumin can reduce NF-κB/NLRP3 signal pathway to inhibit LPS-ATP-induced primary microglial pyroptosis (Ran et al., 2021). In addition to affecting NLRP3 inflammasomes through TLR4/NF-κB pathway, traditional Chinese medicine monomer can also regulate ROS/TXNIP/NLRP3 pathway to prevent pyroptosis. Curcumin (10 μmol L^-1^) inhibits the activation of the TXNIP/NLRP3 pathway by up-regulating AMPK activity in human neuroblastoma SH-SY5Y cells of human neuroblastoma and reducing ROS produced by endoplasmic reticulum stress (Li et al., 2015). Umbelliferone (UMB), a natural antioxidant belonging to coumarin derivatives, is able to cross the blood-brain barrier and protect neuronal cells from death. UMB (15 and 30 mg/kg) pretreatment for 7 days significantly upregulates peroxisome proliferator-activated receptor (PPAR-γ) with an antioxidant effect in MCAO/R rats, and inhibits ROS, thereby reducing the expression of TXNIP to inhibit NLRP3 inflammasome (Wang X et al., 2015). Ginsenoside Rd (Rd), a monomer component of Panax ginseng and Panax notoginseng, is reported to confer neuroprotection in brain injury models. Rd activates the Nrf2 pathway by up-regulating miR-139-5p, and downregulates ROS/TXNIP/NLRP3 induced by MCAO rats and OGD cortical neurons (Yao et al., 2022). Ruscogenin, an important steroid sapogenin derived from Ophiopogon japonicus, has been shown to inhibit cerebral ischemic injury. In the mouse brain microvascular endothelial cells (bEnd.3) and OGD/R primary cortical neuron, ROS was significantly inhibited by the administration of Ruscogenin (Cao et al., 2016). Z-Guggulsterone (Z-GS), an active component derived from myrrh. MCAO rats were treated with different doses of Z-GS. Morphological results showed that Z-GS significantly alleviated neurological deficits, infarct volume and histopathological damage in MCAO rats. It was related to TXNIP and NLRP3 by immunohistochemistry and immunofluorescence staining. The author conducted *in vitro* experiments to verify the mechanism, it was found that Z-GS treatment scarcely changed the expressions of NLRP3 in siRNA-TXNIP pretreated cells compared with the siRNA-TXNIP alone treatment group, suggesting that the neuroprotective effect of Z-GS was dependent on TXNIP-NLRP3 axis (Liu et al., 2020). Traditional Chinese medicine monomer can also upregulate AMPK/Nrf2 and inhibit NLRP3 inflammasome. *In vitro* and *in vivo* experimental studies showed that AMPK/Nrf2 pathway could be downregulated and NLPR3 inflammasome activated in MCAO/R rats or mice, OGD/R or LPS-ATP SH-SY5Y cells, microglial, and astrocytes. When intervening with an alkaloid extracted from Sinomenium acutum, the expression of AMPK was upregulated, and NLRP3 inflammasome was suppressed (Qiu et al., 2016). Astragaloside IV (AS-IV) is the main effective component of Astragalus membranaceus, and is widely used in the prevention and treatment of cerebrovascular diseases (Li S et al., 2021). Xiao et al. (2021) established MCAO/R rat *in vivo*, OGD/R SH-SY5Y cell *in vitro*, they enhanced NLRP3, caspase-1, IL-1β, GSDMD and GSDMD-N protein levels, indicating that NLRP3/caspase-1/GSDMD pathway is activated to promote pyroptosis. AS-IV increased the protein levels of Nrf2 and promoted the transfer of Nrf2 to the nucleus, accelerating Nrf2 activation. It shows that AS-IV inhibits NLRP3-mediated pyroptosis by activating Nrf2 (Xiao et al., 2021). Resveratrol (Res), which is a natural polyphenolic compound, inhibits LPS- and ATP-activated NLRP3 inflammasome and protects microglial cells upon pyroptosis. Mechanismly, it inhibits NF-κB and activates AMPK/Sirt1 pathways (Tufekci et al., 2021). Hispidulin is a flavonoid present in many Chinese herbal medicines (Kut et al., 2022). When MCAO rats were treated with different doses of hispidulin, hispidulin exerted its neuroprotective effects *in vivo* and *in vitro* by suppressing NLRP3-mediated pyroptosis by modulating the AMPK/GSK3β signaling pathway (An et al., 2019). Particularly revealing was that the AMPK inhibitor Compound C (An et al., 2019) or the Nrf2 inhibitor ML385 (Xiao et al., 2021), some of the effects of the drugs are offset, indicating that the traditional Chinese medicine monomers play an anti-pyroptosis effect through AMPK/Nrf2/NLRP3 pathway. Chrysophanol, the main active ingredient from Dahuang (Radix Et Rhizoma Rhei), Heshouwu (Radix Polygoni Multiflori) and Huzhang (Rhizoma Et Radix Polygoni Cuspidwi), can inhibit the activation of NLRP3 induced by transient MCAO (tMCAO) in mice, but the specific pathway mediated by it is not clear at present, which needs to be further explored (Zhang et al., 2014).

Moreover, endothelial cells (ECs) pyroptosis plays an important role in IS. Wang et al. (2021) confirmed that IS can induce pyroptosis of microvascular endothelial cells (ECs) and aggravate the ischemia-reperfusion injury. When MCC950, a specific drug targeting NLRP3, is used to interfere with ECs in OGD, the results show that endothelial NLRP3 is inhibited, indicating that endothelial NLRP3 inflammasome-mediated pyroptosis is also an effective target (Bellut et al., 2021). Some TCM monomers can target ECs pyroptosis. For example, quercetin, luteolin, and epigallocatechin gallate inhibit TXNIP/NLRP3 by reducing ROS in ECs (Wu et al., 2014). Dihydromyricetin (DHM) pretreated vascular ECs, reduced the release of IL-1β related to pyroptosis, significantly decreased the levels of intracellular ROS, and promoted the activation of Nrf2. When the knockdown of Nrf2 by siRNA, the inhibitory effect of DHM on ECs pyroptosis was counteracted. Therefore, DHM plays an anti-pyroptosis role by activating the Nrf2/NLRP3 pathway of vascular ECs (Hu Q et al., 2018) (Table 1).

### Compound prescription and Chinese patent medicine

Due to the occurrence and development of IS is a multi-path and multi-target collaborative process. In the process of treatment, the most effective way is to inhibit or block all the relevant pathways corresponding to the onset of IS. Compound prescription and Chinese patent medicine contain many kinds of effective ingredients of traditional Chinese medicine, which are the most widely used forms of traditional Chinese medicine in clinical practice. At present, studies have confirmed that they can exert neuroprotective effects by affecting pyroptosis. For example, Naoxinqing Capsule, a traditional Chinese patent drug, can effectively inhibit the protein expression of NLRP3, ASC, caspase-1, IL-18 and IL-1β in MCAO/R rats and protect the cerebrovascular function when administered continuously for 21 days at the dose of 100 mg/(kg · d). When OGD PC12 cells were cultured with Shennaofuyuan Decoction (SNFYD) drug-containing serum, the expression level of IL-18 and IL-1β were significantly decreased, suggesting that SNFYD can inhibit neuronal pyroptosis (Li, 2019). Luo et al. (2021) showed for the first time that the expression of TLR4, NLRP3, ASC and caspase-1 was downregulated in a dose-dependent manner after three doses of Angelica Cinnamomum (AC) extract were given to pMCAO rats for 7 days. In LPS-induced BV2 cells, cerebrospinal fluid containing AC extract inhibited the secretion of pro-inflammatory cytokines, and its intervention effect was similar to that of TLR4 siRNA treatment. It suggests that AC may play a neuroprotective role by inhibiting the formation of NLRP3 inflammasome through TLR4/NF-κB. Renshen Shouwu (RSSW), composed of Ginseng (Root of *Panax ginseng C.A. Mey*) and fleece flower root (*Polygonum multiflorum Thunb.*), is a patented Traditional Chinese Medicine included in Chinese Pharmacopoeia. RSSW (50 mg/kg, 100 mg/kg) was administered to MCAO rats. Western blot showed that RSSW significantly downregulated TLR4/NF-κB/NLRP3 signaling pathway (Li. et al., 2020). Taohong Siwu Decoction (THSWD) can also regulate TLR4/NF-κB/NLRP3 (Wang M et al., 2020). THSWD originated from the traditional Chinese medicine book *Yizong Jinjian* of the Qing Dynasty, which is composed of *Prunus persica* (L.) *Batsch*, *Carthamus tinctorius L*, *Angelica sinensis* (Oliv.) Diels, *Rehmannia glutinosa* (Gaertn.) DC., *Ligusticum chuanxiong* Hort, *Paeonia lactiflora* Pall. Also, previous report has investigated the major constituents of THSWD by UPLCQ-TOF-MS. A total of 95 components have been identifified, including aromatic acids, flflavonoids, polysaccharides, volatile oils, monoterpene glycosides, aromatic cyanoglycosides (Dong et al., 2019). They are the basis of THSWD inhibitors of pyroptosis (Ye et al., 2020; Yin et al., 2020). Unlike RSSW, THSWD can reduce the expression of TXNIP, p38MAPK and JNK. In addition, THSWD has been found to inhibit MCAO/R rats pyroptosis via DRP1/NLRP3 pathway (Zhou, 2021). These again illustrate the characteristics of multi-component and multi-target action of traditional Chinese medicine. Panax ginseng and Angelica sinensis (CPA) treatment ameliorated MCAO-induced cerebral damage and neurological dysfunction by inhibiting NLRP3 inflammasome activation and microglial pyroptosis. Its inhibitory effect was comparable to that of MCC950, a well-known inhibitor of NLRP3 inflammasome. Further *in vitro* study revealed that the key active ingredients of Panax ginseng and Angelica sinensis inhibited OGD/R-induced NLRP3 inflammasome activation and pyroptosis by inhibiting DRP1-mediated mitochondrial fission (Hu et al., 2020). Qingkailing (QKL) injection, a patented Chinese medicine approved by the China Food and Drug Administration, has been widely used in clinical practice to treat cerebral ischemia in China. Rats in the QKL group received intraperitoneal injections of 3 mL/kg QKL, QKL relieved IS and suppressed the inflammatory response by inhibiting AMPK-mediated activation of the NLRP3 inflammasome (Ma et al., 2019) (Table 1).

## Conclusion and perspectives

Pyroptosis is a form of death characterized by cell swelling, membrane rupture, and inflammatory cytokine release. It can be caused by NLRP3 inflammasome that assembly after activation of secreted IL-1β and IL-18. NLRP3 inflammasome play an important role in the development of IS. Most *in vitro* and *in vivo* studies in our review have confirmed that inhibition of NLRP3 inflammasome can improve neural function and reduce the volume of cerebral infarction to a large extent. However, a study from *Stroke* showing that the injury degree of IS has nothing to do with NLRP3 inflammasome (Lemarchand et al., 2019). We speculate that this may be related to experimental modeling, because in this report, C57BL/6 mice were modeled with FeCl_3_, which is different from the MCAO and OGD models we summarized previously. A variety of signal molecules lead to early pathological changes through corresponding signaling pathways after IS, subsequently activating NLRP3 inflammasome-mediated pyroptosis. These signal-mediated pathological changes include ROS damage, mitochondrial dysfunction, ion imbalance, lysosomal rupture, and trans-Golgi disintegration. The activation of NLRP3 inflammasome consists of two steps, namely, the activation and assembly of NLRP3 inflammasome. The signaling pathway involved in the activation step is TLR4/NF-κB/NLRP3. The signaling pathways involved in the assembly include ROS/TXNIP/NLRP3, AMPK/Nrf2/NLRP3, DRP1/NLRP3, and TAK1/JNK/NLRP3. Current pharmacological studies show that traditional Chinese medicine can inhibit NLRP3 inflammasome by regulating the above signal pathways, thus inhibiting pyroptosis and achieving the purpose of alleviating the process of IS, showing the characteristics of multi-target and multi-channel treatment of traditional Chinese medicine. This provides a positive signal for exploring the role of traditional Chinese medicine in pyroptosis (Table 1).

We believe that there are still some challenges in the research of anti-IS effect of traditional Chinese medicine based on pyroptosis. 1) Strengthen the research of compound prescription and Chinese patent medicine. Our previous summaries found that compared with traditional Chinese medicine monomer, the compound prescription and Chinese patent medicine, as a common clinical form of traditional Chinese medicine, has less research reports, which is not conducive to providing scientific basis for the clinical application of the compound. In the future, bioinformatics methods can be used to identify the potential effective components and target of action of the compound prescription and Chinese patent medicine, structural pharmacology methods such as molecular docking technology can be used to further reveal the binding sites of monomer components, and methods such as gene knockout or inhibition of key proteins can be used to verify the target of action of monomer components. Adopt modern pharmacological methods to carry out simultaneous component research and comparative study with compound prescription, so as to clarify the material basis of drug function. 2) The IS body model is diversified. Most of the models summarized in this review are MACO or MCAO/R models. However, studies have shown that the selection of models may have an impact on the role of NLRP3-mediated pyroptosis in IS (Lemarchand et al., 2019). Therefore, different models can be used for comparative study in future studies, and mutual verification can fully illustrate the empirical conclusions. 3) To explore the potential of non-classical pyroptosis in the study of IS pathological mechanism. Previous studies have shown that the expression of caspase-11 protein upregulated in OGD microglial cells, which indicates that there is activation of non-classical pyrolytic pathway in the process of IS (Fradejas et al., 2010). However, there is no *in vivo* experiment to show the relationship between non-classical pyrolytic pathway and IS. Moreover, there is little research on non-classical pyroptosis pathway in traditional Chinese medicine.

To sum up, NLPR3 inflammasome may be the switch molecular target of the upstream channel of the mechanism of pyroptosis in IS. Deeply exploring the relevant molecular mechanism of NLRP3 inflammaasome affecting pyroptosis, summarizing the relationship between NLRP3 mediated-pyroptosis signal pathway and IS, and the research status of TCM prevention and treatment, clarifying the specific mechanism of TCM, provide new ideas for TCM treatment of IS, and provide theoretical basis for TCM to effectively and economically serve human health.
